# Highly pathogenic avian influenza A (H5N1) in a backyard flock in Serbia: implications of low biosecurity

**DOI:** 10.3389/fpubh.2026.1920924

**Published:** 2026-09-10

**Authors:** Jelena Maletić, Giulia Graziosi, Jelena Maksimović Zorić, Nemanja Zdravković, Đorđe Aksić, Nemanja Jezimirović, Jasna Kureljušić, Ksenija Nešić, Marco De Nardi, Branislav Kureljušić

**Affiliations:** 1 Institute of Veterinary Medicine of Serbia, Belgrade, Serbia; 2 Department of Veterinary Medical Sciences, University of Bologna, Bologna, Italy

**Keywords:** backyard flock, biosecurity, highly pathogenic avian influenza, One Health, wild-domestic bird interface

## Abstract

In autumn 2024, a highly pathogenic avian influenza (HPAI) H5N1 virus outbreak occurred in a mixed-species backyard poultry flock in central Serbia, comprising hens, ducks, and geese. After the sudden death of two hens, a thorough diagnostic investigation was initiated. Gross examination of the affected hens revealed lesions typical of HPAIV infection, including severe cyanosis of the comb and head skin, and diffuse hemorrhagic lesions of internal organs. On-site investigation included assessment of the remaining birds, environmental sampling, and a biosecurity survey specific to backyard flocks. Although asymptomatic, ducks and geese (*n* = 23) tested positive for avian influenza by both oropharyngeal and cloacal swabs, as well as by serological testing. The biosecurity assessment highlighted major deficiencies, including uncontrolled access to a nearby canal frequented by wild waterfowl. Overall, virus introduction at the domestic-wild bird interface was considered the most epidemiologically plausible hypothesis. However, this pathway could not be confirmed because wild birds were not sampled and viral genome sequencing was not performed.

## 1 Introduction

Avian influenza viruses (AIVs; family *Orthomyxoviridae*, genus *Alphainfluenzavirus*) (1) are highly contagious viruses of birds, with wild waterbirds of the orders Anseriformes and Charadriiformes serving as their natural reservoir (2). Through migratory flyways, AIV-infected birds facilitate the long-distance spread of the virus and contribute to its genetic evolution and genomic reassortment (3–5). Based on their pathogenicity in chickens, AIVs are classified as either low pathogenic or highly pathogenic avian influenza viruses (LPAIVs or HPAIVs). H5 and H7 are the only subtypes associated with HPAI and are subject to notification to the World Organization for Animal Health (WOAH) (6, 7). HPAIVs can cause severe outbreaks with high mortality in both wild birds and poultry (8, 9) with wide-reaching impacts on the poultry industry, public health, the environment, and associated economic sectors (10). Due to their zoonotic potential, HPAIVs also represent a continuous threat to public health (11). Over time, H5 HPAIVs belonging to the Gs/Gd phylogenetic lineage have genetically diversified into numerous clades and subclades. Since 2020, H5 clade 2.3.4.4b viruses have dominated HPAI epidemics, with recurrent epidemic waves occurring across Europe and in Serbia (12). In Serbia, HPAIVs belonging to clade 2.3.4.4b have been repeatedly detected in wild birds since 2016 (13, 14). Most cases have occurred in the northern province of Vojvodina, which contains numerous aquatic ecosystems within the Danube basin and provides important habitats for migratory waterbirds. Mute swans have been among the most frequently affected wild bird species, with recurrent mortality events reported (13, 15). Given the increasing frequency of HPAI detections, a coordinated national surveillance program for avian influenza has been implemented nationwide since 2021. This program combines active and passive surveillance of wild birds and domestic poultry to detect circulating H5 subtype viruses and support timely implementation of control measures (14). Backyard and small-scale poultry production represents an important component of the Serbian poultry sector, accounting for more than half of poultry meat production and approximately two-thirds of egg production (16). Backyard poultry in Serbia is commonly kept under extensive or semi-extensive conditions, including free-range and mixed-confinement systems, in which birds may scavenge freely during the day and are housed in simple or improvised shelters overnight (17). Such flocks may include birds of different species and ages, while outdoor access and limited physical barriers may facilitate contact with wild birds and other animals (17). This interface is particularly relevant to HPAI epidemiology in Serbia. During the 2016/2017 H5N8 epidemic, HPAIV was detected predominantly in wild birds, particularly mute swans, while outbreaks also occurred in backyard poultry holdings characterized by low biosecurity and insufficient protection from contact with wild birds (12). Similarly, during the 2021/2022 epidemiological season, H5N1 infections were detected in both wild birds and backyard poultry, particularly in northern Serbia, a region characterized by numerous aquatic ecosystems supporting migratory bird populations (13). These characteristics highlight the potential epidemiological importance of the wild bird-backyard poultry interface and the need for feasible and effective biosecurity measures adapted to small-scale poultry production systems.

In autumn 2024, sudden mortality involving two hens occurred in a mixed-species backyard flock in central Serbia and was subsequently confirmed as HPAI A(H5N1). A comprehensive outbreak investigation was conducted, integrating pathological examination, molecular and serological diagnostics, environmental sampling, and an on-site assessment of biosecurity practices. This study aimed to characterize the outbreak, investigate potential epidemiological pathways of virus introduction and spread, evaluate biosecurity weaknesses that may have contributed to transmission, and discuss practical measures for HPAI prevention and control in backyard poultry systems within a One Health framework.

## 2 Materials and methods

### 2.1 Diagnostic investigation

A backyard mixed-species flock consisting of two adult hens, six geese, and 17 ducks (*n* = 25) was investigated after the owner reported the sudden death of both hens in late October 2024. Both carcasses were submitted to the Scientific Institute of Veterinary Medicine of Serbia (Belgrade) for necropsy and diagnostic investigation. Based on the gross pathological findings observed during necropsy, HPAI was immediately suspected and pooled tissue samples (spleen, kidneys, lungs, pancreas, and intestines) were collected for molecular testing. A suspension was prepared from 1 cm^3^ of different tissues, homogenized with a pestle and mortar, and diluted 1:10 in Dulbecco’s Modified Eagle Medium (DMEM, Thermo Fisher Scientific, United States), supplemented with antibiotics and antimycotics (MycoZap, Lonza Bioscience, Switzerland). The homogenate was further centrifuged at 1500 g for 10 min. Extraction of viral RNA from the obtained supernatant was performed with the IndiSpin Pathogen Kit (Indical Bioscience GmbH, Germany). The influenza A virus genome was detected using the Luna® Universal Probe One-Step RT-qPCR Kit (New England BioLabs, England) with previously published primers and probes (32).

Initial detection of influenza A virus RNA was performed at the Scientific Institute of Veterinary Medicine of Serbia. Confirmation of the H5N1 subtype and high pathogenicity was subsequently performed at the Serbian National Reference Laboratory for Avian Influenza (Veterinary Specialized Institute “Kraljevo”) using tissue samples collected from the two hens.

Following laboratory confirmation, an epizootiological investigation was carried out at the backyard holding. Following written approval of the Veterinary Inspector, in compliance with appropriate biosecurity measures to reduce transmission and spread of HPAI, individual oropharyngeal and cloacal swabs and blood samples from the wing vein were collected from the remaining geese and ducks. Fresh feces were also obtained from the environment, and water samples from the drinking containers. All swabs, fecal, and water samples were tested for the presence of the influenza A virus genome using the previously described real-time RT-PCR protocol. In the laboratory, swabs were soaked in 1 mL of PBS, homogenized for 2 min at 50 Hz, and centrifuged at 4000 rpm for 5 min. Fecal samples were prepared as a 10% suspension in PBS, homogenized, and centrifuged together with water samples at 8000 rpm for 20 min. Supernatants were used for RNA extraction and downstream RT-PCR. All samples with Ct below 40 were evaluated as positive. A serological investigation was performed on 23 blood samples using an indirect ELISA to detect and quantify antibodies against the nucleoprotein of influenza A virus (ID Screen Influenza A Nucleoprotein Indirect ELISA, ID Vet, France). According to the manufacturer’s instructions, titers >668 were interpreted as positive.

### 2.2 Biosecurity assessment

A biosecurity assessment of the backyard flock was conducted during the on-site epidemiological investigation following laboratory confirmation of HPAI. For this purpose, the standardized Biocheck. UGent questionnaire, specifically developed for backyard and small-scale poultry production, was used (15). The questionnaire evaluates external and internal biosecurity through questions covering the introduction and movement of poultry, feed and water supply, manure and carcass management, visitors and farm personnel, infrastructure and biological vectors, farm location, disease management, and cleaning and disinfection practices. The assessment generates biosecurity scores ranging from 0 to 100, with higher scores indicating better implementation of biosecurity measures.

The questionnaire was administered by members of the investigation team through a structured interview with the flock owner, who responded to the individual questions. The responses were recorded by the investigators and, whenever applicable, simultaneously verified through direct observation of the holding, poultry housing, surrounding environment, and management practices. Any discrepancies between the owner’s responses and the conditions observed during the on-site assessment were clarified with the owner and recorded according to the observed situation. The original Biocheck. UGent questionnaire was used without modification. Before the assessment, the owner was informed of the study objectives and provided written informed consent.

## 3 Results

### 3.1 Diagnostic investigation

The flock originally consisted of two adult hens, six geese, and 17 ducks (*n* = 25). The backyard holding was located on the outskirts of Belgrade, with no other poultry holdings within a 20 km radius. The outbreak was first recognized following the sudden death of the two hens, which represented the only birds showing clinical disease. The remaining six geese and 17 ducks showed no clinical signs throughout the investigation. After laboratory confirmation of HPAI in the hens, an official epidemiological investigation was initiated by the Veterinary Authority. Oropharyngeal and cloacal swabs, blood samples from all remaining birds, together with environmental fecal and drinking water samples, were collected for laboratory analysis.

**Figure 1:**
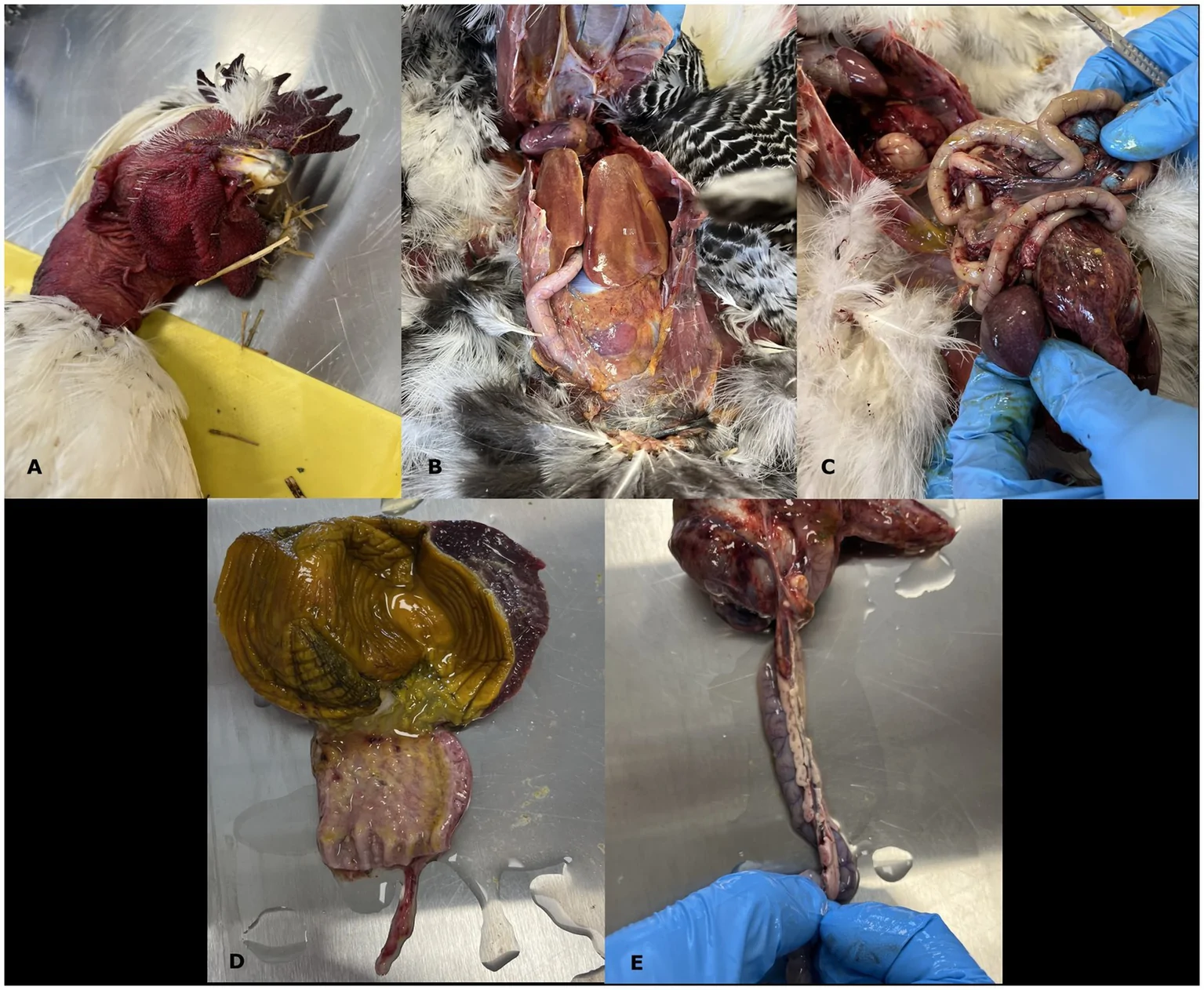
Gross pathomorphology findings: Comb and skin cyanosis **(A)**, celomic cavity petechiae **(B)**, necrotic foci on the enlarged spleen **(C)**, proventriculus–ventriculus junction and proventricular mucosa with petechial and ecchymotic hemorrhages **(D)**, and multifocal necrotic areas with hemorrhages **(E)**.

Gross examination of the two hens revealed multiple lesions typical of HPAIV infection. The most obvious external finding was severe cyanosis of the comb, wattles, and head skin, giving the birds a dark bluish-to-purple coloration (Figure 1A). At the opening of the coelomic cavity, petechiae on the coelomic fat were observed (Figure 1B). The spleen was enlarged and firm, with multiple pale to yellow-gray necrotic foci, indicating lymphoid depletion and necrosis (Figure 1C). Petechial and ecchymotic hemorrhages were observed on the mucosa of the proventriculus and at the proventriculus–ventriculus junction (Figure 1D). The pancreas contained multifocal necrotic areas with associated hemorrhages, consistent with virus-induced necrotizing pancreatitis (Figure 1E).

The results of the molecular and serological investigations are summarized in Table 1. Highly pathogenic avian influenza A(H5N1) virus was confirmed in tissue samples collected from the two hens. All individual oropharyngeal (6 geese and 17 ducks, *n* = 23) and cloacal swabs (*n* = 23), as well as pooled fecal and drinking water samples, tested positive for influenza A virus RNA. The RT-qPCR Ct values obtained from pooled oropharyngeal and cloacal swab samples, fecal samples, and drinking water samples ranged from 32.7 to 36.5. In addition, all 23 serum samples were positive for antibodies against influenza A virus, with antibody titers exceeding the diagnostic threshold.

**Table 1:** Diagnostic investigation performed at the backyard flock.

Sample source	Type of samples	Positivity rate (%)	Result
Hens	Tissue samples (pooled)	2/2 (100)	HPAI^†^ A(H5N1)
Geese and ducks	Oropharyngeal swabs	23/23 (100)	IAV^‡^
	Cloacal swabs	23/23 (100)	IAV
	Fecal samples (pooled)	1/1 (100)	IAV
Environment	Drinking water samples	2/2 (100)	IAV

^†^HPAI, highly pathogenic avian influenza; ^‡^IAV, Influenza A virus.

### 3.2 Biosecurity assessment

The biosecurity assessment of the backyard holding is presented in Table 2. According to the Biocheck. UGent questionnaire, the overall biosecurity score was 49%, with external biosecurity (53%) scoring higher than internal biosecurity (34%).

**Table 2:** Biosecurity evaluation of the backyard flock using Biocheck.

Biosecurity category	Score (%)
Purchase of eggs/day-old chicks	n.a.†
Purchase of laying hens	19
Depopulation and transport	100
Feed and water supply	64
Manure and carcass removal	49
Visitors and farm personnel	48
Infrastructure and biological vectors	18
Location of the farm	71
External biosecurity (overall)	53
Disease management	17
Cleaning and disinfection	46
Internal biosecurity (overall)	34
Total biosecurity score	49

^†^Not applicable. UGent system for poultry backyard/small-scale farms.

**Figure 2:**
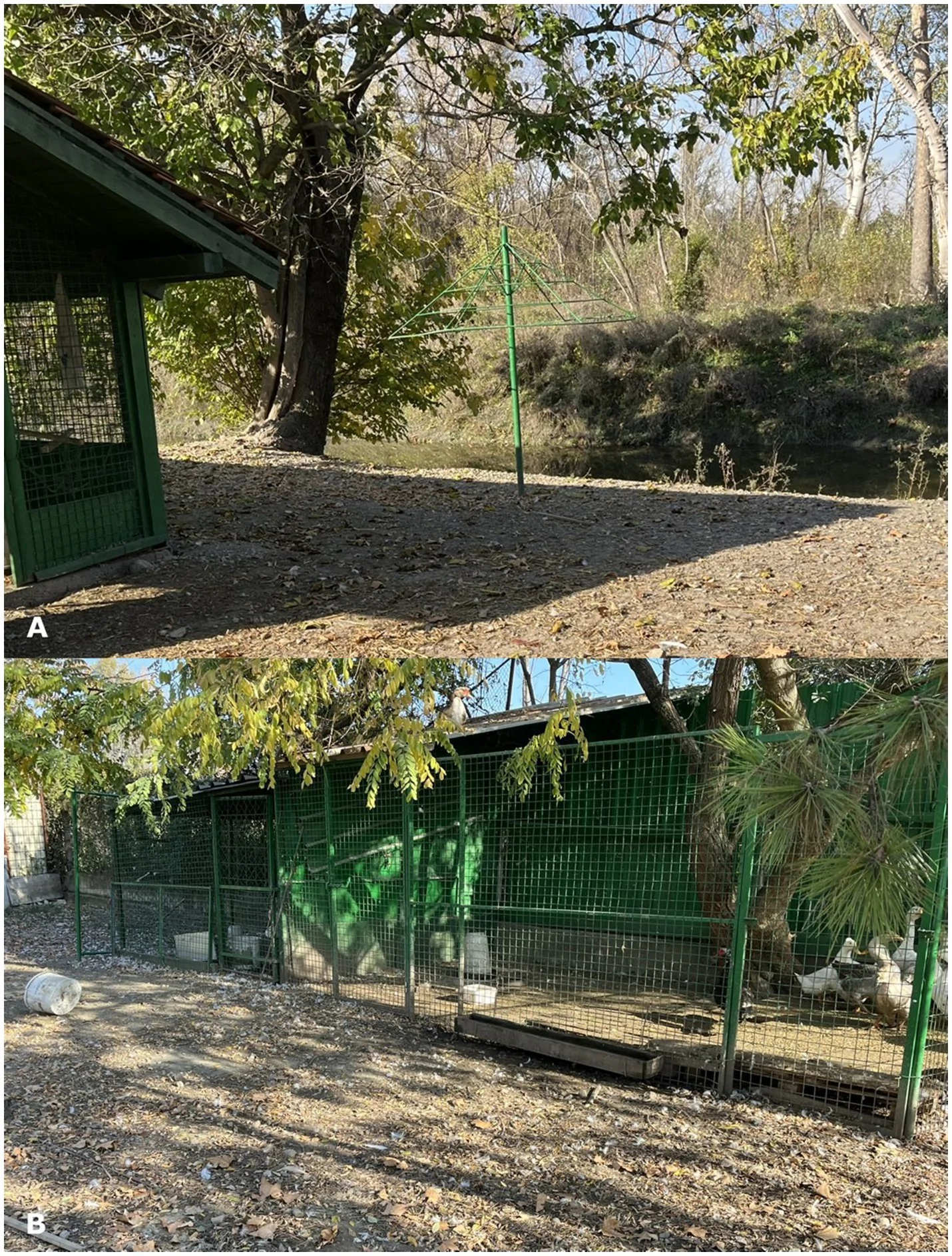
The flock confinement system allowed limited outdoor access within netted enclosures, with access to the nearby canal **(A)**. Different age categories within multiple species were housed together **(B)**.

The flock was managed under a mixed-confinement system. The holding consisted of several poultry shelters, each associated with a small fenced outdoor enclosure, within the larger perimeter of the property (Figure 2A). The fences surrounding the individual outdoor enclosures were relatively low and did not completely prevent birds from leaving the enclosed areas. A water channel bordered part of the holding and was frequented by wild waterfowl. Along this section, no perimeter fence separated the holding from the channel, creating a potential interface between domestic poultry and the surrounding aquatic environment. However, direct contact between domestic birds and wild waterfowl was not observed or documented during the investigation. Multiple poultry species were housed together, and different age categories were present within the flock (Figure 2B).

Among the external biosecurity measures assessed, laying hens were purchased from different suppliers, and transport of newly purchased birds to the holding was not consistently direct, with stops at other facilities depending on the suppliers’ delivery routes. Furthermore, the owner was unaware whether the transport equipment had been cleaned and disinfected before delivery. Newly introduced birds were not isolated before being mixed with the existing flock. Although no live birds or eggs were sold and farm equipment was not shared with other holdings, the birds were fed a combination of commercial poultry feed and mixed grains. Feed was stored in bags in a designated storage area; however, storage conditions did not completely prevent potential exposure to moisture, wild birds, or rodents. Cats, dogs, and vermin occasionally entered the poultry areas, representing additional potential pathways for pathogen introduction.

With respect to internal biosecurity, the lowest score was attributed to disease management (Table 2). Veterinary supervision was minimal or absent, and no vaccination against common poultry diseases (e.g., Newcastle disease, infectious bronchitis, or infectious bursal disease) was implemented. Sick birds were only occasionally separated from healthy individuals. Drinking water and feeding systems were cleaned irregularly and rarely disinfected. In addition, procedures for the handling and disposal of dead birds were inconsistent.

### 3.3 Control measures

**Figure 3:**
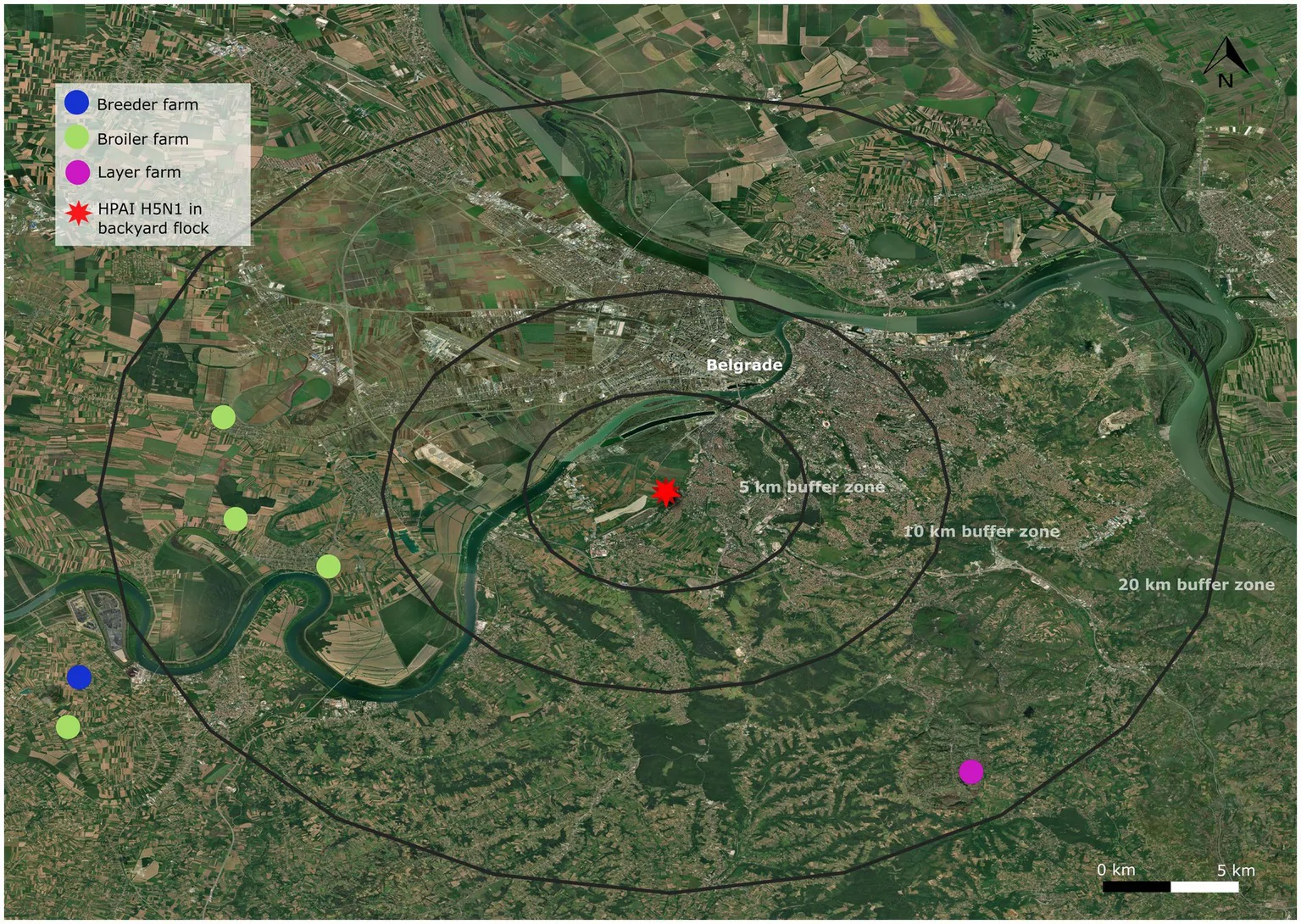
Situational awareness with the HPAI-infected housing and distance from nearby commercial farms.

Control measures were implemented in accordance with national legislation (18). As the holding consisted of a backyard flock with non-commercial birds, the Veterinary Authority granted a derogation from the measures applied to commercial poultry. This decision was based on the absence of clinical signs in geese and ducks, as well as the location of the holding, which was situated between 10 and >20 km from the nearest commercial poultry farms (Figure 3).

All birds were allowed to remain alive, but were restricted from leaving the facility and were kept indoors. The entire holding underwent thorough disinfection using a broad-spectrum virucidal disinfectant. To decrease the risk of zoonotic disease transmission from infected birds, good hygiene practices (e.g., hand-washing with warm, soapy water after handling birds or their excrement) and the use of personal protective equipment (e.g., dedicated clothing and footwear, airway protection when in proximity to birds) were implemented. Respiratory and cloacal swabs from geese and ducks were collected 21 days after the initial HPAI detection and retested. All resulted negative for IAV (data not shown).

## 4 Discussion

This study reports an HPAI A(H5N1) outbreak in a mixed-species backyard flock on the outskirts of Belgrade during autumn 2024. Typical HPAIV-associated pathological lesions were observed in the two hens, in which the highly pathogenic avian influenza A(H5N1) virus was confirmed. All sampled geese and ducks tested positive by both molecular and serological assays despite remaining clinically healthy throughout the investigation, indicating widespread intra-flock transmission. This finding is epidemiologically relevant because waterfowl may remain asymptomatic after H5N1 infection, potentially allowing viral circulation to go undetected in mixed-species backyard flocks. Recent modeling of H5N1 transmission in small-scale mixed-species backyard flocks further suggests that the presence of ducks may delay outbreak detection and increase the undetected burden of infection, particularly when infected ducks remain asymptomatic (19).

Based on the results of the epizootiological investigation, contact at the domestic-wild bird interface was considered the most epidemiologically plausible pathway for HPAIV introduction. Among the biosecurity weaknesses identified, the location of the holding adjacent to a water channel frequented by wild waterfowl represented a particularly relevant risk factor. Although the geese and ducks were kept in small fenced outdoor enclosures adjacent to their shelters, the enclosure fences were relatively low and did not completely prevent the birds from leaving the enclosed areas. Moreover, the water channel bordered part of the holding where no perimeter fence was present, creating a potential opportunity for direct or indirect contact between domestic poultry and wild waterfowl or their contaminated environment. Given the established role of wild waterbirds in HPAIV ecology and previous HPAI detections in wild birds in Serbia (10, 12, 20), this interface was considered the most plausible epidemiological pathway for virus introduction. Other identified biosecurity deficiencies, including the introduction of birds from multiple sources without quarantine, inadequate protection of feed, and unrestricted access of cats, dogs, and vermin to poultry areas, represented alternative potential pathways of virus introduction. Feed contamination was considered as another potential pathway, as the flock received both commercial poultry feed and mixed grains that were stored in bags. However, the ability of AIV to remain infectious in feed is influenced by the characteristics of the feed matrix, and different feed materials may therefore present different transmission risks (21). Where feed is considered a plausible transmission pathway, appropriate treatment or disinfection procedures may therefore contribute to reducing the risk of virus transmission (21). As neither the feed nor the storage environment was tested for HPAIV, the contribution of contaminated feed to virus introduction could not be determined. The potential role of rodents should also be interpreted cautiously. Experimental challenge studies have demonstrated the susceptibility of synanthropic rodents to H5N1 HPAIV but found no evidence of viral shedding in feces, suggesting that fecal contamination by infected rodents may be an unlikely transmission pathway (22). Nevertheless, rodent-mediated transmission cannot be completely excluded, as the virus has been detected in respiratory samples and/or tissues following experimental infection (22, 23). However, direct contact between domestic and wild birds was not documented, and in the absence of wild-bird sampling, viral whole-genome sequencing, and detailed tracing of bird movements, the relative contribution of these potential pathways could not be determined. Therefore, the introduction of HPAIV from wild birds remains an epidemiologically plausible hypothesis, but it could not be confirmed.

Previous HPAI outbreaks in Serbia have involved backyard poultry, including H5N8 in 2016/2017 and H5N1 in 2022, and similar patterns have been observed elsewhere, underscoring the epidemiological relevance of backyard production systems in HPAI epidemics (24, 25). Biosecurity represents a key component of HPAI prevention in backyard and small-scale poultry holdings (26), although relatively few studies have addressed on-site biosecurity assessment in these production systems (25–27). The biosecurity profile of the investigated flock, with an external score of 53% and an internal score of 34%, aligns with previous observations in backyard poultry systems, where disease management, animal introduction practices, and prevention of contact with wild birds have been identified as important biosecurity weaknesses (15, 27, 28).

Major biosecurity gaps identified during the present investigation included inadequate disease management, insufficient visitor and personnel control, and poor cleaning and disinfection practices. Weaknesses in external biosecurity included the proximity of an accessible water source frequented by wild birds, the purchase of birds from multiple suppliers without consistent transport hygiene or quarantine, inadequate protection of feed, and unrestricted access of cats, dogs, and vermin to poultry areas. Internal biosecurity was also poor, with minimal veterinary supervision, inconsistent separation of sick birds, irregular cleaning and disinfection, and improper carcass disposal. Together, these deficiencies may have provided multiple opportunities for HPAIV introduction and subsequent within-flock transmission.

Implementing comprehensive biosecurity and disease control measures can be particularly challenging in backyard poultry systems (26, 29). Therefore, biosecurity recommendations for these holdings should focus on measures that are feasible and adapted to local production conditions. Based on the findings of the present investigation, priority measures should include restricting poultry access to open water sources frequented by wild birds, strengthening physical barriers to minimize contact with wildlife and other animals, protecting feed and drinking water from contamination, implementing quarantine for newly introduced birds, improving carcass disposal and cleaning and disinfection practices, and promoting routine veterinary supervision and owner education. In the specific setting investigated here, improving perimeter fencing along the water channel and increasing the height or effectiveness of fences surrounding outdoor poultry enclosures would represent practical measures to reduce opportunities for contact between domestic poultry and wild birds or potentially contaminated aquatic environments. Although complete separation of domestic poultry from wild birds may not always be feasible in backyard production systems, targeted biosecurity measures adapted to the specific risk profile of individual holdings may help reduce opportunities for HPAIV introduction and spread (26).

From a One Health perspective, this outbreak highlights the importance of integrated surveillance of poultry, wild birds, and individuals who may have been exposed to infected animals. The absence of clinical signs in infected geese and ducks in the present outbreak also highlights a potential limitation of surveillance approaches that rely predominantly on clinical signs or mortality, particularly in mixed-species backyard flocks. Species-specific differences in clinical expression and infection dynamics should therefore be considered when designing surveillance strategies for backyard poultry systems (19). Backyard flock owners and veterinarians play key roles in the early detection and reporting of suspected HPAI cases, while the appropriate use of personal protective equipment and hygiene measures remains essential to reduce zoonotic risk during outbreak investigation and response (10, 11). The close interface among domestic poultry, wild birds, other animals, and humans in backyard production systems further emphasizes the need for collaboration between veterinary, wildlife, and public health sectors in HPAI preparedness, surveillance, and control (10, 11).

Regarding outbreak management, several control measures were implemented following HPAI confirmation, including restriction of bird movements, indoor confinement of the remaining flock, thorough disinfection of the holding, and reinforcement of hygiene and personal protective measures. Follow-up RT-qPCR testing of the remaining geese and ducks 21 days after the initial HPAI detection yielded negative results. Although the contribution of individual interventions cannot be assessed in this single-flock observational investigation, the absence of further clinical disease and negative follow-up testing suggest that movement restrictions, confinement, disinfection, and reinforced biosecurity measures may have contributed to limiting further virus circulation within the holding. The decision to keep the remaining birds alive, rather than apply measures typically implemented in commercial poultry holdings, was made by the Veterinary Authority based on the specific epidemiological circumstances of the holding, including the absence of nearby commercial poultry farms. Considering the perspectives of backyard flock owners and their understanding of HPAI control measures is important when implementing disease control strategies in non-commercial holdings (30, 31). Control strategies should therefore combine adequate disease containment with clear communication and practical guidance adapted to backyard poultry keepers, which may facilitate cooperation with veterinary authorities and timely reporting of suspected cases.

This investigation has several limitations. First, it describes a single backyard flock, limiting the generalizability of the findings. Second, wild birds from the surrounding environment were not sampled; therefore, the presumed source of virus introduction could not be confirmed. Third, whole-genome viral sequencing and phylogenetic analyses were not performed, preventing genetic comparison with viruses circulating in wild birds or associated with other poultry outbreaks. Furthermore, direct contact between domestic poultry and wild birds was not documented, and because several control measures were implemented simultaneously, the effectiveness of individual measures could not be evaluated. Consequently, the proposed route of virus introduction remains epidemiologically plausible but cannot be confirmed directly, and the effectiveness of specific control measures cannot be inferred from this single outbreak investigation. Nevertheless, the pathological, molecular, serological, environmental, epidemiological, and biosecurity findings provide valuable insight into HPAI occurrence and management in backyard poultry and support the implementation of integrated surveillance and feasible, risk-based biosecurity measures within a One Health framework.

## 5 Conclusion

Clear guidance provided by the Veterinary Authority and private veterinarians, supported by practical educational materials and affordable biosecurity measures, may improve compliance with good farming practices and reduce disease transmission at the backyard poultry-wild bird interface, thereby protecting both animal and public health.

## Data Availability

The original contributions presented in the study are included in the article/supplementary material, further inquiries can be directed to the corresponding author.
